# Developing behavioural activation for people with acquired brain injury: a qualitative interpretive description study of barriers and facilitators to activity engagement

**DOI:** 10.1186/s40359-023-01230-2

**Published:** 2023-07-13

**Authors:** Andrea Kusec, Abigail Methley, Fionnuala C. Murphy, Polly V. Peers, Estela Carmona, Tom Manly

**Affiliations:** 1 MRC Cognition and Brain Sciences Unit, University of Cambridge, 15 Chaucer Road, Cambridge, CB2 7EF UK; 2 Department of Experimental Psychology, University of Oxford, Radcliffe Observatory Quarter, Anna Watts Building, Oxford, OX2 6GG UK; 3 Innovative Clinical Psychology Solutions Ltd, London, W1W 5PF UK

**Keywords:** Acquired brain injury, Stroke, Brain tumour, Traumatic brain injury, Behavioural activation, Qualitative, Depression

## Abstract

**Background:**

Acquired brain injuries (ABI) from stroke, head injury, or resected brain tumours are associated with poor emotional wellbeing and heightened risk of mood disorder. Common sequalae of ABI, such as poor attention and memory, can create barriers to the efficacy of cognitively demanding mood interventions, such as Cognitive Behavioural Therapy (CBT). Behavioural Activation (BA), where individuals plan and engage in reinforcing activities, is a promising alternative due to lower cognitive demands. However, BA was initially developed in clinical populations without ABI where the primary barriers to activity engagement were low mood and anxious avoidance. Additionally, BA can incorporate a range of techniques (e.g., mood monitoring, activity scheduling, targeting avoidance, contingency management) and psychoeducational topics (e.g., mindfulness, managing uncertainty; social/communication skills). Exploring barriers and facilitators to adopting specific BA components in ABI is an important aim.

**Methods:**

Semi-structured interviews were conducted with purposively selected ABI survivors (*N* = 16) with both low and high depressive symptoms, and family members (*N* = 7). Questions focused on routine and enjoyable activities, and feedback on 10 different BA techniques and associated psychoeducational topics. Transcripts were analysed using an interpretive description framework. Analysis was informed by field notes, reflexivity diaries, and peer debriefing.

**Results:**

The final constructed framework, *Creating Sustainable Engagement,* comprises a two-tier hierarchy. Higher-level themes concerned core perspectives of BA, regardless of BA component discussed. This included identifying optimal time windows for different BA components (*Right Tool at the Right Time*)*,* that BA components should, at least initially, not be burdensome or fatiguing (*Perceived Effort*), that emotional readiness to confront activity-mood relationships should be addressed (*Emotional Impact*)*,* and that planned BA activities be consistent with individual values (*Relation to Values*)*.* Lower-level themes concerned specific BA components: Of these, activity scheduling, procedures targeting avoidance, managing uncertainty and social/communication skills were generally well-received, while mood monitoring, contingency management, and mindfulness had mixed feedback.

**Conclusions:**

BA is a widely scalable intervention that can be adapted for ABI. This study provides a novel framework on implementing a range of BA components in ABI and adds to the limited evidence on which components may be particularly suitable.

**Supplementary Information:**

The online version contains supplementary material available at 10.1186/s40359-023-01230-2.

## Background

Individuals with an acquired brain injury (ABI) are at increased risk for depression. Causes of ABI include head trauma (traumatic brain injury; TBI), an interruption to the brain’s blood or oxygen supply (stroke, anoxia) or brain tumours [1]. Notably, up to half of adults with TBI, brain tumour, or stroke will experience clinical depression within 5 years of their injury, a markedly increased risk compared to the non-ABI population [2–4]. Emotional outcomes post-ABI are further complicated by common impairments such as poor memory and attention, physical disability, and fatigue [5, 6]. Such impairments can lead to increased dependence on family, often necessitating family involvement in post-ABI treatment [7–9].

Unfortunately, emotional support needs of those with ABI are often not met. For example, two thirds of people with a stroke requiring emotional support feel that their needs are not met [10]. In TBI, psychological services are the least frequently accessed, and are least likely to be offered compared to physical and cognitive rehabilitation [11]. Similarly, adults with brain tumours with greater emotional distress report requiring more care needs compared to those with lower emotional distress [12]. Psychological therapies of known efficacy in non-ABI populations, such as Cognitive Behavioural Therapy (CBT), have mixed results in ABI [13–15]. This may be attributable to CBT’s relatively high demand on cognitive capacities such as memory, processing speed, language and abstract reasoning, all of which can vary widely in ABI [15–17].

Behavioural Activation (BA) [18, 19], a mood intervention developed in non-ABI populations, is a promising alternative in ABI. BA theory stems from the observation that low mood tends to be associated with reduced activity levels. Reduced activity may be due to lack of energy, poor motivation, low expectations about the outcomes of activities, and/or avoidance due to fear of failure and negative consequences. While restricting activity level may seem protective and conserve energy, the BA approach argues that the consequent reduction in positive experiences and reinforcement actually lowers mood further. This, in turn, further diminishes motivation and increases avoidance. BA aims to reverse this vicious cycle by encouraging “activation” through engagement in enjoyable activities. The relative simplicity of BA makes it suitable for a wide range of people. Meta-analyses of BA for depression support its efficacy as being on par with CBT and antidepressants in those without ABI [20]. BA has been further demonstrated to enhance subjective wellbeing [21, 22], with increased activity level associated with greater life satisfaction [21] and positive affect [23].

In stroke and TBI, many studies have documented correlations between activity levels and mood [24–28]. However, beyond changes in mood, there are many important contributors to decreased activity level in ABI. These can include interruption to work and study, diminished mobility, reduced income, social isolation, fatigue, pain, lowered self-confidence, and cognitive impairments. Encouragingly, there is evidence that despite these additional contributors, BA may be suitable in ABI. In a randomised trial, Thomas et al. [29] provided BA to adults with stroke with language impairments and low mood. Compared to those receiving usual care, participants receiving BA were noted to have improved mood as rated by their family members. Further support of BA in ABI was demonstrated by Thomas et al. [30]—in a feasibility study, adults with stroke with depression were offered one-to-one BA (mean 8.5 sessions). Relative to those receiving treatment as usual, the BA group showed clinically meaningful reductions in depression, and the intervention was perceived as acceptable and feasible. These studies provide promising initial evidence of BA in mood management following ABI.

An attractive feature of BA is that it can be delivered effectively without extensive therapeutic training [30, 31]. However, there are many variants of BA. Aside from “hallmark” BA techniques such as activity scheduling and mood monitoring, BA can include additional components such as goals and values assessments, relaxation training (e.g., progressive muscle relaxation), contingency management (e.g., self-reward procedures), procedures targeting verbal behaviour (e.g., positive reframing), and procedures targeting avoidance (e.g., identifying and mitigating avoidance patterns). Longer courses of BA have included psychoeducational topics such as mindfulness, communication skills, and social skills to facilitate greater activity engagement [32]. There are good theoretical reasons why each of these components could be helpful in BA. However, if they are not perceived as helpful, tolerable or feasible by an individual, the chances of engagement or regular use of a component may be reduced. For example, while Thomas et al. [30] found that most experienced improvement following BA, some showed no change or worsened. Whilst it is difficult to rule out factors in individuals’ lives beyond the BA intervention that may account for positive and negative outcomes, it is possible that different BA components could have been more acceptable/effective.

Although therapists are trained to deliver BA in a person-centred way, many BA therapists may be less familiar with some issues faced by adults with ABI that could impact benefit from BA. In a mixed-methods review of knowledge of ABI among UK health care professionals, there was a general lack of understanding of ABI from acute care to community-based services [33]. Given the potential of BA for to help manage the increased risk of mood disturbance in ABI, and the benefits of increased knowledge of how therapists can support clients with an ABI, the current study aimed to explore experiences of activity engagement and perceptions of BA components in ABI.

Here, we utilise qualitative methodology to obtain an in-depth understanding of contributors to activity levels and perceptions of BA components from both those living with an ABI and their family members. Specifically, this study uses interpretive description methodology, a constructivist approach to qualitative data to that is often employed develop practical information for health care settings [34]. Importantly, interpretive description aims to capture subjective experiences of individuals whilst framing researcher experience and knowledge as useful in analysing the data. We aim to provide information that may be useful to therapists working with individuals with an ABI and to inform the development of BA interventions that may be suitable for those with an ABI.

### Study purpose

The purpose of this study is to explore perceptions of adults with ABI and their family members on 1) barriers to engaging in meaningful or enjoyable activities; 2) facilitators of such engagement, and 3) the likely utility of specific BA techniques and psychoeducational topics in fostering greater engagement. A secondary purpose of this study is to use study responses to inform the development of the treatment manual of a BA intervention in ABI [35].

## Methods

### Research team characteristics

The research team consisted of neuropsychological rehabilitation and mental health specialists. AK has experience conducting qualitative research in ABI [36] including a Master’s degree in Rehabilitation Science. AM and TM are registered clinical psychologists. AM works in a clinical neuropsychology service, with doctoral training in qualitative research in neurological populations. EC has experience with community ABI populations and a Master’s degree in clinical neuropsychology and cognitive neuroscience. Authors FCM, PVP and TM have doctoral degrees in cognitive neuropsychology and extensive experience working with ABI populations and intervention development. The authors believe, based on available evidence and personal experience, that a reduced amount of reinforcing activities levels contribute to low mood and depression, and that increasing engagement in positively reinforcing activities contributes to a positive mood.

### Ethical approval

This study was given ethical approval from the University of Cambridge Psychology Research Ethics Board Committee (2018.059) and was preregistered on August 18^th^ 2018 [37].

### Interview development

AK developed the semi-structured interview based on the Kanter et al. review [32] of BA components and BA clinical guides [38], BA theory [18, 19], self-determination theory [39], as well as personal and supervisory team experience of working with neurological populations [40–42].

The interview covered experiences of everyday activities, such as hobbies and socialising. We additionally included questions on the helpfulness of 10 different components that may be used in BA (see Table 1 for included components; refer to Results for definitions). For a review of each component, see Kanter et al. [32]. Two additional components concerned a) the balance between activities seen as predominantly functional (e.g., grocery shopping, chores) and activities where reward or satisfaction are the main aims (e.g., hobbies, entertainment, socialising), and b) managing uncertainty in planning activities. These components were included based on the relationship of both routine and enjoyable activities to mood [43], the robust association of intolerance of uncertainty to mood [44], as well as personal interest. This interview was developed specifically for this study, and a copy is in the Supplementary Materials.

**Table 1:** Interview topics and example questions for each category. See http://osf.io/btwg3 for full interview script

Topic	Example Question
*Experiences in Day-to-Day Activities*	“We are going to talk about day-to-day activities, such as chores, work, appointments, and so on. Can you tell me what gets you motivated to complete these activities?”
*Experiences in Enjoyable Activities*	“Would you say that taking part in enjoyable or pleasant activities is important to you? Why or why not?”
*BA Techniques* *Mood Monitoring* *Activity Scheduling* *Active Engagement* *Alternative Solutions* *Self-Rewards* *BA Psychoeducational Topics* *Social Skills* *Communication Skills* *Mindfulness* *Routine vs. Enjoyable Activities* *Managing Uncertainty*	“Some people find it useful to keep a diary and plan in pleasant activities to look forward to. Do you think this would be helpful for you? Why or why not?” (*Activity Scheduling*)

### Participant recruitment

We invited selected members of the Cambridge Cognitive Neuroscience Research Panel (CCNRP) to participate. The CCNRP is a panel of adults with chronic ABIs due to focal lesions, comprising predominantly adults with stroke and resected brain tumours recruited via review of radiological scans who have expressed interest in participating in research. CCNRP members had previously completed a short battery of cognitive and emotional measures that were available for this study.

We purposively sampled CCNRP members based on Patient Health Questionnaire-9 scores (PHQ-9 [45]), a brief depression measure. For purposive recruitment aims, participants were categorised as either “no low mood” (< 5 symptoms on the PHQ-9) or “low mood” (> 4 symptoms present plus at least depressed mood/anhedonia present; see Fann et al. [46]). The aim was to increase the variety of possible combinations of activity level to mood to explore a range of experiences. Additional inclusion criteria were 1) age over 18, 2) ability to speak and comprehend English, and 3) sufficient capacity to consent to, and tolerate, the interview (based on previous CCNRP evaluation). Our aim was to interview an approximately equal number of men and women. Once recruited, we asked participants whether an adult family member (e.g., partner, child, sibling) could be approached for a separate interview for data triangulation to enhance data trustworthiness. Not having such a person, or not consenting to their contact, did not preclude participation. For family members that were approached, we asked them to provide feedback on each interview topic with regards to the individual with the ABI.

### Interviews

After providing informed consent, AK conducted one-to-one interviews either in a private testing space within the University, the participant’s or family member’s home, or over the telephone, depending on individual preference and circumstance.

Interviews took approximately 30 to 60 min and were audio recorded with participant consent. Participants selected pseudonyms for the interviews which are used to present the results. Prompts were used to encourage elaboration and clarifying remarks (e.g., “What I am hearing is that you have a hard time beginning an activity – is this correct?”). Field notes were taken throughout to capture non-verbal and contextual information. Reflexive journaling, where AK reviewed the interview and potential conceptualisations of the data, was completed within 24 h of interview.

The interview was iteratively developed throughout data collection and analysis to refine interview questions. Commonly, qualitative research concludes once saturation is reached (i.e., when there are no new themes in the data). However, the assumption that one can know all aspects of an area of inquiry in a single study, and hence reach saturation, has been challenged in interpretive description research [34]. Thus, our aims were pragmatic in interviewing enough participants to reach data saturation, rather than theoretical saturation, to build a practical framework that may have utility in increasing activity levels in ABI.

### Data analysis

**Fig. 1:**
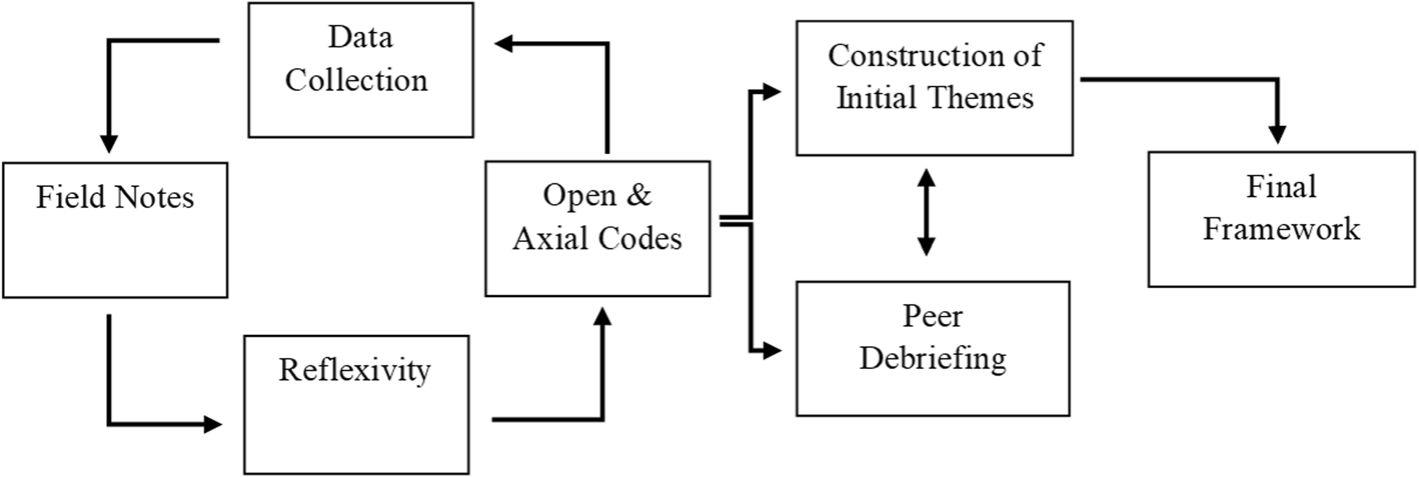
Analysis procedure. Upon finishing data collection, field notes were written. Reflexivity, completed continuously throughout analysis, is an evaluation of beliefs and conceptualisations of the data and interview process. Open codes refer to the smallest discrete unit of data from transcripts that are provided a label. Axial codes refer to categories labelled by linking open codes together. We then further linked these themes into a construction of themes, used for reviewing the data with peers (peer debriefing)

AK transcribed all participant interviews, and family member interviews were transcribed by EC. Personally identifiable information was removed upon transcription. Analyses were conducted using the constant comparative method [47], a process of concurrent data collection and analysis. Within interpretive description, the interviewer’s knowledge and experiences are thought to provide valuable insight to the data, thus analysis primarily being conducted independently is typical [34, 48]. The data analysis process is presented in Fig. 1. Data interpretation was inductive, that is, giving precedence to participant responses irrespective of our views on their merits. Early stages of analysis focused heavily on big picture questions such as “What am I learning about this?” [34]. Transcript data were sorted into discrete categories using *open coding*, consisting of initial labelling of transcript text (e.g., “planning as a motivator”). Potential links were then considered between all open codes and collapsed these into broader categories, a process termed *axial coding*. Referring back to the transcripts, field notes, and reflexive journal entries, these axial codes were then further refined and constructed into an initial *interpretive thematic summary* [34]. Throughout analysis, the social skills and communication skills questions were found to have elicited similar responses from participants, and subsequently resulted in similar construction of themes. Hence, we analysed them as a single component.

Authors AK, FCM, PVP, TM and AM engaged in peer debriefing sessions and discussed collective impressions of the initial thematic summary and review of transcripts from those with and without self-reported depressive symptoms. Feedback was incorporated into subsequent analyses by considering their fit within the initially constructed themes, re-examining the transcripts, and reconstructing the framework. It was agreed that greater description in the results would be of more use to therapists when delivering BA in ABI than combining the data into a smaller number of themes. Thus, we decided to present barriers and facilitators per BA component and construct central themes that relate to activity levels overall. The final framework received general acceptance.

## Results

The constructed dataset supporting the conclusions of this article, as well as the interview script, is available in the Open Science Framework repository, available at http://osf.io/btwg3.

Sixteen participants and 7 family members participated in this study (see Table 2 for full details). Participants varied widely in education, ABI aetiology, and occupational status, however there was no diversity in self-reported ethnicity.

**Table 2:** Participant characteristics

**ABI Demographic Information (*N* = 16)**		**Min—Max**
**Gender**		
Man	9	
Woman	7	
**Age** – M (*SD*)	62.25 (10.61)	47 – 78
**Ethnicity**		
White British	16	
**Highest Level of Education**		
Primary School	1	
GCSE/O-Level	5	
A-Level	1	
Undergraduate Degree	3	
Postgraduate Degree	6	
**Aetiology**		
Meningioma	6	
Stroke	5	
Other brain tumour	4	
Brain scarring due to head trauma	1	
**Current Living Situation**		
Living with Spouse	8	
Living with Family	8	
**Current Work Status**		
Retired	7	
Working full-time	4	
Working part-time	4	
Unemployed	1	
**PHQ-9 Status**		
Low Mood	7	
Without Low Mood	9	
**ABI Severity – EBIQ-S** – M (*SD*)		
Total Score (possible range 63 – 189)	94.62 (21.40)	66 – 132
Cognitive (possible range 12 – 36)	18.38 (4.66)	12 – 31
Physical (possible range 5 – 15)	6.38 (1.78)	5 – 11
Communication (possible range 4 – 12)	5.88 (1.67)	4 – 9
**Family Members (*N* = 7)**		
**Gender**		
Man	2	
Woman	5	
**Age** – M (*SD*)	55.17 (20.51)	19 – 73
**Ethnicity**		
White British	7	
**Relationship to Participant**		
Husband	1	
Wife	4	
Adult Child	2	

*EBIQ-S* European Brain Injury Questionnaire-Self

### Framework for development of BA in ABI

The following sections present the constructed framework for considering themes relevant to developing BA for adults with ABI. The first section, entitled *Creating Sustainable Engagement,* comprises four central themes. The second section addresses barriers and facilitators to BA components.

### Creating Sustainable Engagement

**Fig. 2:**
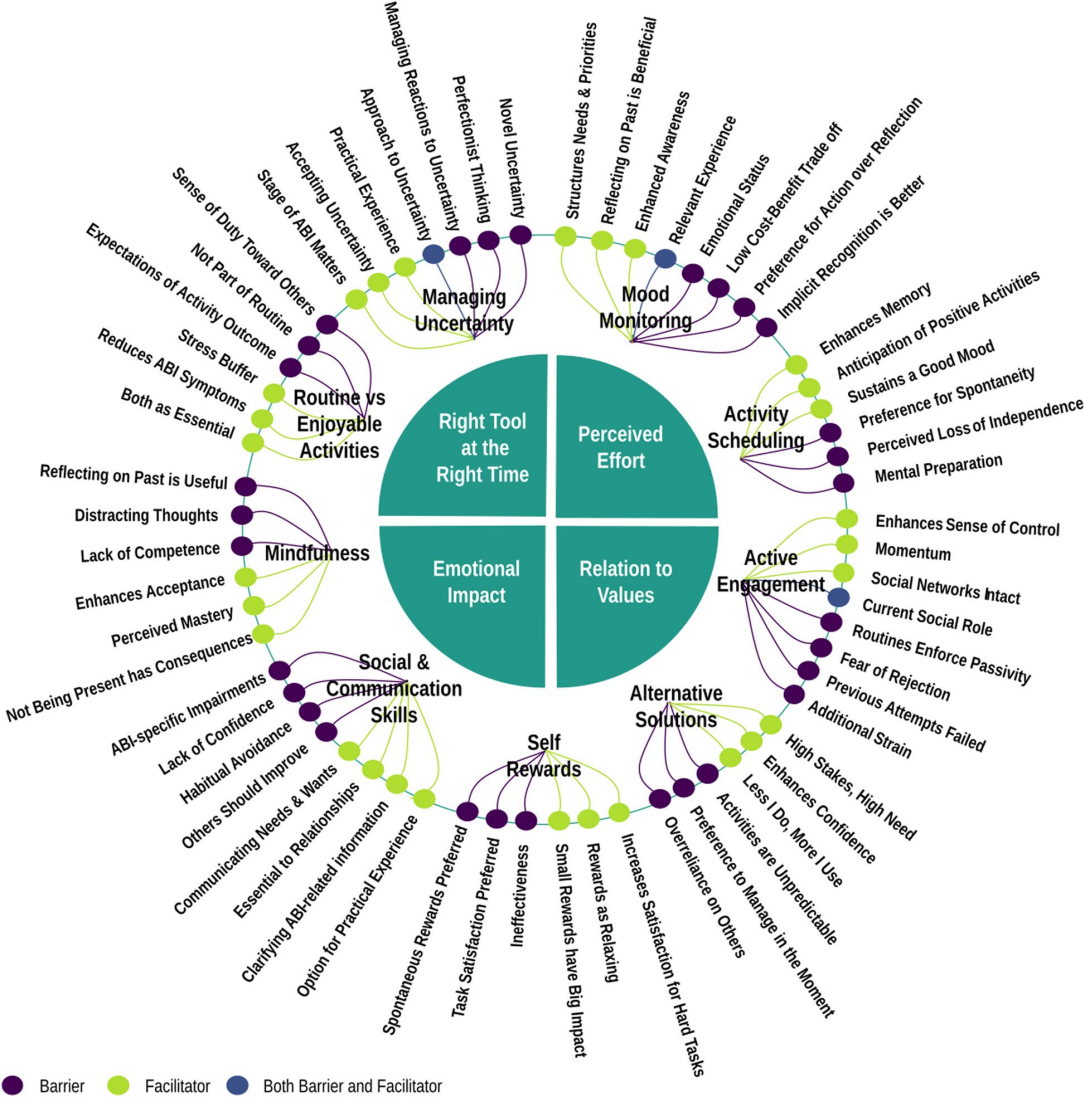
The final constructed framework entitled Creating Sustainable Engagement based on participant interviews. The central quadrants represent the four core themes of the framework, with component-specific (e.g., mood monitoring, activity scheduling) facilitators and barriers in the outer circle. When considering how to create engagement in activities that will continue beyond therapy time, it may be useful to consider the fit between particular BA components and the stage of the client’s ABI (*The Right Tool at the Right Time*); individual differences in perceptions of how effortful an activity is based on current capabilities (*Perceived Effort*), what emotional responses the client may have to BA (*Emotional Impact*), and how therapeutic targets link to the client values (*Relation to Values*). Component-specific facilitators and barriers are considered secondary to the central quadrants in considering ways to deliver BA therapy. Figure available under CC-BY 4.0 license [34]

It was apparent that interview responses were not just about the activities or components themselves but rather person-specific aspects that could be barriers or facilitators to activity engagement. ABI in particular is associated with highly variable outcomes in severity and effects of ABI (such as poor memory), opportunities to engage in activities (e.g., access to transport), support and encouragement from family, and other factors such as having dependents, all of which may affect decision-making in planning activities. Thus, participants often focused on factors that could sustainably support activity engagement rather than those that may be more transient. Example quotes of themes and subthemes are in Table 3, with the framework visualised in Fig. 2.

**Table 3:** Four central themes of *Creating Sustainable Engagement* and its subthemes. All subthemes are illustrated by relevant participant quotes

Theme	Subtheme	Example Quotes
**Right Tool at The Right Time**	Is This Tool For Me	*“It doesn’t matter to me where I improve [my communication skills] or not. I don’t need it at my age.”* Participant Cory
	Coping with Early Stages	*“At the time [of his stroke], he was very frustrated with not being able to do things himself but he also would blame mum for a whole lot of things”* Family Member Rose
	Chronic Changes to Abilities	*“I did get diagnosed with chronic fatigue last year…I kind of get to about 11 o’clock, 12 o’clock and I just hit a brick wall and I just can’t function.”* Participant Linda
**Perceived Effort**	Willingness to Work for Others	*“[The activity] is for somebody else. So I’m prepared to get up at silly o’clock and get myself organised.”* Participant Charles
	Resisting Passivity	*“I might meet somebody, and then it’ll become nothing after that. I will not follow it through and I might even not keep in touch with some friends with whom I should have. And then friendships would die. And I know I need to make an effort but that effort to maintain is hard.”* Participant Alice
	Success Sustains Effort	*“I think [completing an activity] kind of gives you a spur – you know that you’ve achieved something and therefore you can go forward and do it again.”* Participant David
	Increased Prioritising and Planning	*“I do need to sleep in the afternoon, so therefore I would go from one to five, which [activity] is more important?”* Participant Sophia
	Activities as a Habit	*“Well, you have your routine and it has to be done. He’s very strict with the routine.”* Family Member Sandy
**Emotional Impact**	Managing Self-Criticism	*“Occasionally [I’m] self-critical that I think things have not worked out the way I want them to. Sometimes I think that things will work out better than they do, and you know sometimes they just don’t. And I do think then that it’s usually, I think I must have done something wrong, to have done that, but really it should have worked but it didn’t”* Participant Kelly
	Recognising How Activities Affect Me	*“[Enjoyable activities] lift my heart. Life, for everybody regardless of your circumstances is full of challenges, and some of those are difficult and wear you down. So I need, I need those sort of therapeutic uplifts.”* Participant Bella
	Gaining Confidence	*“I’m always very wary [about trying new things]. I just don’t know enough about it, it worries me.”* Participant James
	Coping with Failed Activities	*“If [an activity] doesn’t work out we can find something else. I’m very much a ‘Let’s not worry about it’ person.”* Participant Sophia
	Aversion to Confronting Emotions	*“For [communication skills], I don’t like arguments and everything and I get upset easily”* Participant Bonnie
	Changes in Social Circle	*“I said to the members of my family, I said ‘look, you’ve got to appreciate that someone in my position, it’s too much expecting me to do everything’…think about things that I might want doing.”* Participant Merlin
	External Praise	*“You have to encourage him all the time…’you’ve made a lovely job with that, well done!’ That kind of thing.”* Family Member Sandy
**Relation to Values**	I Do What I Value	*“If it’s anything to do with family, that comes first.”* Participant Roy
	New Perspective on Life	*“Since I had my brain tumour…nothing really bothers me that much. I just take every day as it comes…you know people would understand what I’m saying, who’s actually been through the same thing, they would know exactly 100 percent”* Participant Cory
	Closeness to Others	*“Everybody when you get older have got their own jobs on their own with their own family their own way. When we were single we had loads of time to say hello to people. We didn’t have [time] so much anymore and so it has be certain people, you know. And so there is the little key nucleus.”* Participant Sophia

### Sustainable Engagement – the Right Tool at the Right Time

An important theme was whether it was the “Right Time” for participants to increase activity level. Time-related barriers included age (e.g., “too late in life to change”), occupational status (“no time”) and perceived social roles (e.g., caretaking responsibility). Importantly, the “Right Time” was not linearly related to time post-ABI, especially in terms of confronting emotional challenges. Though emotional responses to the ABI were perceived as more intense in its immediate impact on e.g., abilities, in chronic ABI the focus of emotional consequences shifted to the ABI’s impact on family members, social and occupational life, and adapting to chronic changes.

Whether or not a BA component was the “Right Tool” was based on preference, experience, and whether it was manageable given person-specific post-ABI physical and cognitive ability – simply not perceiving the need or relevance for the BA component could influence its perceived worth in the long-term.

“*I’ve never even thought about [scheduling something enjoyable] …we keep ourselves busy, I wouldn’t think that would do anything.*” (Participant 8, Fred).

Some described how the early stages of ABI could be emotionally distressing, and formerly “easy” tasks were now difficult with the onset of new impairments including both physical changes such as difficulties walking, and cognitive changes such as taking longer to process new information – as such, there was little scope for thinking about increasing activity level. Establishing new habits was seen as time consuming, and could disrupt routine, increase risks (e.g., injury, fatigue) and expose limitations due to ABI such as speech issues or slowed information processing. Family members generally were hesitant about attempts to increase activities very early post-ABI, with some components perceived as irrelevant.

Facilitators to increasing activity engagement included recognising how all types of impairments affected activity levels, wanting to exceed expectations of others, and regularly engaging in activities to maintain recovery. Simpler BA components were thought as especially valuable in early stages of ABI.

“*[Mood monitoring is] such a simple thing…during the early stages [of my ABI], I think I could have done with the simplicity of that sort of structure.*” (Participant 9, Bella).

### Sustainable Engagement – Perceived Effort

A recurring barrier to increasing activity level was the amount of effort required; a limited commodity perhaps better expended on necessary activities. Far more than simply physical effort, participants more often described requiring more mental effort to plan, organise, and execute steps needed for daily activities. As a result, participants reported engaging in greater prioritisation and planning of activities, and were less likely to complete activities perceived to be too difficult or irrelevant. One participant described needing time for mental preparation prior to a task.

“*I have to, I have to know what I’m doing…I have to know in advance so that I can get it clear in my head what I’m doing.*” (Participant 15, Linda).

For highly valued activities, expending limited effort was worthwhile, especially if others were involved. Some wished to reduce *wasted* effort, making sure that invested energy paid off. For example, when discussing an upcoming trip, a participant said:

“*I want to achieve what I set out to do that day. So it is important to plan. I don’t want to be left in a situation where I arrive and there’s nothing happening. It’s not every day we’re doing things.*” (Participant 7, Roy).

Completing an activity helped motivate effort for other activities. As one participant noted “*Once you’re successful in one thing then you’re feeling ‘well okay I’ll try other things as well’”* (Participant 2, Sophia). The subjective effort required to engage in new activities diminished once they became habitual.

### Sustainable Engagement – Emotional Impact

Participants highlighted a variety of emotional responses to activities and BA components. Recognition of the positive emotions associated with enjoyable activities could, of course, be a strong facilitator.

“*I can’t imagine how miserable life would be without [enjoyable activities]. It really would be miserable without them. If they didn’t happen I think I’d just go downhill, curl up, and die.*” (Participant 7, Roy).

Others, however, struggled with self-critical thoughts, and felt frustrated if ABI impairments, such as poor memory, interfered with activities. Low confidence became a barrier and negatively affected mood. Changes to relationships after ABI, and the emotional responses of others, affected activity levels.

“*I got to the point where my physical lack of ability to do all these things was such that … my wife was no longer enjoying what was going on, and she was concerned, frightened beyond belief that I was going to do something stupid and I did a couple of times. And I thought, I had to just think about it for the weekend and I said to my wife ‘I don’t think this is going to work for me, I’m going to give it up’…I feel that I’m too much of a burden upon these, the staff that are running [the swimming programme]*” (Participant 13, Charles).

*Anticipated* emotional responses acted as a barrier; when discussing mood monitoring, one participant felt that rating how activities made her feel would be distressing.

“*I have to live with myself every day and I don’t want to write [my mood] down, I would have to see it.*” (Participant 15, Linda).

Successfully overcoming self-critical thoughts, including not perceiving a failed activity to have negative implications and retaining confidence, were facilitators to sustaining activities. Others benefitted from external praise.

### Sustainable Engagement – Relation to Values

This theme represents how an activity relates to the values of the individual. For some, simply enjoying an activity was perceived as valuable. Others placed value on experimentation with new activities. Some placed greater emphasis on satisfaction, or even sense of duty, for activities completed for others, regardless of how enjoyable the activity was perceived to be. For those with a brain tumour, surviving a potentially life-threatening event and the potential for recurrence seemed to prompt critical reflection on valued aspects of one’s life, with these individuals often noting that they have been “*given a second chance at life*.” This acted as a facilitator to engagement and enhanced positive reinforcement from daily activities. When describing his mother, one family member noted:

“*She can’t know that [her brain tumour] won’t take a turn for the worse, and you know unless she’s had an MRI yesterday she could wake up tomorrow and she could be close to death. I think she has an amazing ability to put that aside, and I think her faith helps with that…it allows her to keep a positive mindset and she feels she’s doing her best.*” (Family Member 1, Clark).

The activities in which participants sustained engagement were ones they valued most. In particular, a sense of belonging and closeness to others, as well as a desire to be understood and appreciated, acted as facilitators.

“*I feel alive, I feel who I am [when I do something enjoyable]. I feel that other people need me as much as I need them. I feel like I can do something for other people and make them happy.*” (Participant 11, Alice).

### Barriers and facilitators for specific BA components

The second set of themes presents responses to BA components; in particular, aspects that may be a barrier or facilitator to their adoption. As most participants had no direct experience of BA, these are anticipated responses based on similar experience, self-knowledge, and speculation on how they are likely to respond to the proposed components.

### BA technique – Mood Monitoring

Mood monitoring requires individuals to think about and record how they feel before, during, and/or after activities. It is a technique designed to increase awareness of the links between activities and reinforcement. Facilitators included recognising that this enhanced awareness, using mood monitoring to provide structure and evaluate needs and priorities, and evaluating whether novel activities were mood-enhancing.

“*I think [mood monitoring] might help him generally assess areas in his life and help him focus on the things that do improve his mood.*” (Family Member 6, Rose).

Others, though recognising its value, perceived regular monitoring as too effortful to maintain, or felt resistant to exploring how activities made them feel. Some preferred implicitly evaluating the activity-mood relationship rather than explicitly writing it down. Mood monitoring was viewed as unnecessary when in a good mood, and may become aversive in a low mood. Some had experience with similar formats of monitoring activities, which were either a barrier or facilitator.

“*I tried to keep a happiness diary. I kept going for a week and then I stopped. Because it simply meant I had to discipline myself into doing yet another activity at the end of each day… as long as I later became aware of these things and just, stopping and thinking ‘Aha, I’m having a happy moment now’ I don’t feel the need to write it down.*” (Participant 11, Alice).

There was a general sense from participants and family members that they were already aware of what they enjoy, and hence mood monitoring was at best irrelevant, and at worst patronising and “*a total waste of time”* (Family Member 3, Sandy).

### BA technique – Activity Scheduling

In activity scheduling, clients are encouraged to identify and schedule potentially mood-enhancing activities, the premise being that explicit plans are more likely to be enacted. This rationale was broadly accepted, with facilitators including memory benefits and sustaining a good mood. Participants emphasised the importance of having something to look forward to, and mitigating stress.

“*Absolutely useful…it restrains stress levels, and you just get out and do something.*” (Participant 16, Bonnie).

Barriers highlighted by participants were related to physical, cognitive, and financial constraints, rather than scheduling per se*.* Consequently, some felt they had reduced independence. Needing a sense of “readiness” was a barrier, especially for something unexpected or complex. Some expressed a distaste for planning activities in advance and preferred engaging in activities spontaneously. This related both to the effort involved in planning, and uncertainty about how they would feel the day of the activity.

“*I just like to take things as they come naturally…say you block out Saturday morning to do something in particular. I don’t like to do that because I might not feel like doing that on the day, you see. So, for example, this weekend my husband wanted to go to Chilton, and we were planning, but now, I don’t feel like doing it now, so we’re not going. I don’t feel things are set in stone and I have to do them.*” (Participant 14, Adriana).

### BA technique – Active Engagement

BA treatment manuals (e.g., Martell et al. [38]) distinguish between the *process* of engaging in a potentially reinforcing activity and the activity itself, in particular whether the activity was initiated by the individual – termed here as “active engagement.” Participants felt that initiative and agency were important, yet expressed difficulties in reducing passivity. An important facilitator was the desire to actively contribute to wellbeing rather than be a passive recipient of care.

“*When I was first diagnosed one thing that really frustrated me was that I was given no options for how I could help myself. I was just told to watch and wait, and that wasn’t good enough for me. I needed to be doing something. And all the medical people I spoke to, including a GP at the surgery I went to, told me to just keep up with the drugs and that would be enough. And that wasn’t enough. I needed to actually be involved in trying to make myself better.*” (Participant 9, Bella).

Once an activity was successfully initiated, the resultant “momentum” was a facilitator to sustain initiation for further activities. For some, especially in early stages of ABI, the input of family and friends helped maintain this momentum in activity levels, easing the transition to subsequently greater self-initiation.

Some participants noted previous attempts to self-initiate had gone poorly, or had concerns that self-initiating would be too straining. A fear of rejection from others when initiating social activities was a barrier.

“*I’m not very good at [taking initiative] and sometimes I don’t call friends enough. I kind of sit back and wait for them to call me which I know is wrong…there’s always a slight doubt in your mind…sometimes you’ll be rebuffed or that person will not be as enthusiastic as you are and therefore you kind of feel you shouldn’t*” (Participant 1, David).

Perceived social role could be a barrier or facilitator, with some family members stating it was their role to initiate activities rather than the participant’s.

### BA technique – Alternative Solutions

One technique to reduce avoidance involves generating alternative solutions in anticipation of barriers that may arise to a planned activity. The perceived value of this technique depended on the complexity and/or rarity of the activity, involvement of others, and any potential consequences of failure. Alternative solutions were thought to increase confidence in executing plans, and provide reassurance.

“*Only for complicated things…where other people are involved in the end result. If it was just me and I was making a very complicated meal and it went wrong I’d just say ‘I’ll do without it then’ but I put more expectations on myself if I’m doing something for somebody else and that’s when I need that plan.*” (Participant 6, Kelly).

However, some participants felt that generating alternative solutions was not always a good use of time. Given the inherent unpredictability of future activities, some expressed a preference for managing barriers as they occur, rather than in advance. For others, generating alternative solutions was the role of family members, not the person with the ABI.

“*It would be good to think of that, but as I say I have [my wife] to help me do that. I don’t have to think that way.*” (Participant 13, Charles).

“*He really wouldn’t worry about it…. that’d be me who’d panic.*” (Family Member 3, Sandy).

### BA technique – Self-Rewards

Whilst BA generally encourages engagement in activities that are inherently rewarding, additional rewards can be used to supplement reinforcement. Participants felt that self-rewards increased task satisfaction, particularly for complicated or difficult activities, or found small self-rewards relaxing. Some viewed self-rewards as essential to daily life.

“*I believe it’s really important that we all learn to congratulate ourselves. Even just, and I say this to my students a lot, you know you work hard at something and you see yourself achieve something and you must when you get to the end say ‘Well done me!’ and give yourself a pat on the back because we live in a world that doesn’t encourage that. I think they’re essential to everything, and there shouldn’t be a single day that goes by without some sort of self-praise or reward.*” (Participant 9, Bella).

Others were divided over the use of self-rewards, and found them too indulgent or preferred task satisfaction.

“*I don’t think I’m that sort of person. It just doesn’t appeal to me…the amount of effort and time you put into something is a reward in itself without any sort of tangible thing at the end.*” (Participant 1, David).

Others felt that self-rewards could be ineffective or overused, and preferred to have spontaneous celebration of achievements.

### Psychoeducational topic—Social & Communication skills

Longer courses of BA have included skills training to foster positive relationships and maintain social reinforcement [32]. Participants acknowledged the importance of social and communication skills, especially in communicating personal needs and wants to others, but perceived them as difficult to develop. In particular, effectively conveying ABI-related information was a facilitator.

“*People make assumptions if you don’t communicate things…and they can treat you in a different way when they, if they think they’re making allowances for you or whatever. I mean it was definitely difficult telling people about my, my meningioma. I was doing a ‘wait and see’ and see what happened with it and there didn’t seem to be any point in telling my children because it would have worried them. But I did learn from that, I think you have got to talk to people about health problems*” (Participant 6, Kelly).

Participants stated that practicing difficult social situations and creating positive social atmospheres were desirable. However, implementing these skills were thought to be hindered by ABI-related impairments such as speech difficulties, habitual avoidance of social situations, and decreased confidence when socialising with others.

“*I don’t believe in myself that I can. I’m so concerned that I can’t, um, put words in my mouth properly like I used to.*” (Participant 16, Bonnie).

A notable barrier was feeling that others should improve their social and communication skills instead of themselves, specifically when inquiring about ABI impairments. For example, when describing her eye being permanently shut following her ABI, one participant stated “*an issue is I meet new people and they’re focusing on my eye rather than me. I’d rather people asked me about it than just look at me”* (Participant 15, Linda).

### Psychoeducational topical – Mindfulness

Borrowing from mindfulness-based therapies, BA has encouraged individuals to stay “in the moment” when engaging in activities to enhance reinforcement. Participants generally interpreted staying “in the moment” as a general philosophy rather than a specific “experience savouring” technique. Some found this perspective useful to enhance acceptance of post-injury life, and reduce focus on concerns about how they might change as a consequence of their ABI in the future. When asked why staying in the moment was important for her, one participant stated:

“*Well, it’s, you can’t change the past. And there’s no point in revisiting that because you can’t change it. And the future, well nobody knows what going to happen from one day to the next. Just because you’ve had a brain injury, that’s, you know, I don’t believe anything to do with my brain is any more likely to happen to me than anybody else.*” (Participant 6, Kelly).

Some saw the merits of mindfulness, but were unsure of how to implement it or became easily distracted by negative thoughts. Some were *not* receptive to mindfulness, as they found it beneficial to reflect on the past.

“*It’s just, it’s quite important, it’s quite important to think of life, any mistakes previously so that you don’t actually repeat them.*” (Participant 10, Cory).

### Psychoeducational topic – Balancing routine vs Enjoyable activities

Within BA, the level of positive reinforcement that activities afford is important [38]. Although “routine” activities can be reinforcing (e.g., high level of mastery), here we use “routine vs enjoyable” as shorthand for activities that are perceived as repetitive or arduous but necessary, and activities where there is an explicit expectation of enjoyment. Like self-rewards, beliefs around dedicated time for enjoyment seemed to affect responses. Some found this “balance” to act as a “stress buffer” and helped manage the effects of an ABI, while others viewed regular engagement in both types of activities as essential.

“*Every individual has got their own life to live. And I feel that my life is unique, and I want to live it on my terms. There are certain things to me that are important to me and I need to make space for those things in my life. It’s essential for me to do things that I enjoy, as well as responsibilities that other people need.*” (Participant 11, Alice).

Some had difficulties prioritising enjoyable activities, especially if there were many competing routine demands or if they had expectations of the outcomes of activities that were not met. Others implied that seeking out personal enjoyment would be not be a useful habit to develop, or that a duty toward others was more important.

“*I don’t have time for relaxation…everybody else comes before I do. So I have to make sure that my son is catered for, cooked for, clean, all the rest of it. And then my husband, I have to sort his tea and what have you. I don’t give a thought to me as long as everybody else is happy*.” (Participant 15, Linda).

“*I do think [planning both types of activities] would be helpful. But I keep referring back to [my wife] because she does everything. So I’m very fortunate I don’t have to worry about things like that.*” (Participant 13, Charles).

### Psychoeducational topic – Managing Uncertainty

There is evidence linking “intolerance of uncertainty”, or difficulties managing uncertainty, to depression [44]. Increasing activity levels may result in increases in uncertainty, therefore an important element of BA may be managing responses to uncertainty. Facilitators to learning how to manage uncertainty included being in early stages of ABI, and an awareness of the futility of entirely predicting the future. Having support from others who have experienced similar health difficulties was viewed as helpful.

“*It would have been helpful to have had some help with that…in a normal life if you’re not challenged by an illness you still have those uncertainties and you work out ways, or you get help working out ways to deal with them. When an illness, a serious illness is thrown at you often times you can’t get that help from your friends or your family because they haven’t experienced what you’re going through*.” (Participant 9, Bella).

Participants had distinct approaches to uncertainty, such as becoming fatalistic about their future, relying on religious faith to cope with uncertainty, or concerns about situation-specific, rather than general, uncertainty. These approaches were either a barrier or facilitator. Barriers included finding the sudden increases in uncertainty post-ABI too overwhelming to manage, negative reactions to uncertainty such as fears of injuring oneself again, and perfectionist thinking.

“*For the brain injury, wondering what that really means, is that event going to occur again, and what really controls that event, and why did it happen in the first place? And how, what influences it. You know certain things influence it, you need to take your medication, control your blood pressure, but then how do you control your blood pressure? And is the medication really doing what it should do? There’s all those sorts of aspects to it. So I think there is an anxiety associated with having a stroke that I never really thought of before I had it.*” (Participant 1, David).

## Discussion

The purpose of this study was to explore experiences of activity engagement in ABI, and identify barriers and facilitators to adopting potential BA components. In this study, there was variability in responses driven by individual differences in abilities, beliefs, experiences, and contexts. The framework *Creating Sustainable Engagement* posits that, given changes in abilities, social circumstances, and employment, supporting each individual in how to generate habitual and ongoing activities that provide reinforcement in light of these is likely to be important to success within BA therapy with adults with ABI.

### Right Tool at the Right Time, Perceived Effort, Emotional Impact, and Relation to Values

The theme Right Tool at the Right Time is perhaps unsurprising given variable outcomes in activity levels post-ABI [49]. For example, only half of individuals with TBI return to pre-injury leisure activities, even at 10 years post-injury [27], and a median of up to 68% of community-dwelling stroke survivors report requiring support in social participation up to 4 years post-stroke [50]. Similarly, 45% of adults with brain tumours report persistent difficulties in participation at 2 years post-diagnosis [51]. This suggests the “Right Time” to intervene may not uniquely occur in early stages of ABI. Beyond preference, selecting the “Right Tool” (and increasing activity engagement more broadly) was linked to the physical and cognitive effort (both perceived and actual) required to execute it. Consistent with recommendations to modify CBT in ABI [16], there was a view that simpler techniques would have been especially preferable in early ABI. However, participants noted that if an activity became habitual, it required less mental effort. In BA for ABI, encouraging habitual, highly valued activities that can become part of an individual’s routine may result in lasting mood benefits, especially for those with more severe cognitive impairment. There is evidence that recurrent, compared with one-off activities, had greater success in BA case studies with adults with TBI and stroke [52]. Of course, given that the sample was in chronic stages of their ABI, any ongoing impairments may have influenced perceptions of BA components and therefore their adoption.

The “Right Time” likely does not only relate to amount of time post-injury but a readiness to confront emotional difficulties, increasing activity level, and navigating changes to social relationships – something participants both with and without low mood found challenging. In a study investigating changes in competency post-TBI, van Walsem and colleagues [53] reported that participants felt they most lacked competency in emotional and cognitive capabilities. The “Right Time” may therefore be when one moves beyond the more immediate physical challenges to recognising there is a “gap” in one’s skillset, or when an individual acknowledges the impact of emotional changes on one’s life Positively, many participants recognised the benefit of enjoyable activities and how a lack thereof would be detrimental. However, those with low mood may especially struggle with maladaptive coping responses to failed activities [54] and may find therapy aversive. Indeed, in our data greater self-reported emotional distress aligned with greater mention of self-criticism and perceiving failed activities as a personal failure. It may be useful to align BA with Acceptance and Commitment Therapy approaches and evaluate whether encouraging planning in *valued* activities can overcome maladaptive coping patterns in BA, rather than encouraging planning *enjoyable* activities.

### Participant views on BA components

The literature on efficacy of BA in ABI is limited, and even further so, the suitability and preference of BA components. In a systematic review [55] only four studies explored use of BA in ABI, and only in adults with stroke and TBI. The components included psychoeducation for carers, mood monitoring and activity scheduling, graded task assignment and problem solving. With the exception of activity scheduling, Oates and colleagues [55] have not reported on the specific success of each component, nor acceptability of components from participants, in part due to inconsistencies in reporting how content was adapted for ABI and lack of data on fidelity assessments of what was delivered.

### BA techniques: Mood Monitoring & Activity Scheduling

Mood monitoring and activity scheduling are hallmarks of BA, often included in brief BA therapy [56]. Conceptually, both techniques were perceived as useful by participants, but there were challenges that it could be too effortful, unnecessary, and/or counterproductive. Similarly, in Thomas and colleagues’ BA trial in stroke [30], activity scheduling was perceived to be valuable but there was mixed feedback on mood monitoring, with some valuing its reflective purpose but others finding it repetitive or difficult. Although the degree to which mood monitoring’s effectiveness relies on explicit versus implicit recognition is yet to be determined, caution is warranted as expectations of use may differ from practical experience – especially as, outside of ABI, mood monitoring on its own can decrease depressive symptoms and increase activity [57–59]. The less-than-optimal feedback to mood monitoring could be due to those with low mood finding it aversive or too effortful, while for those without low mood, explicit monitoring provides no new information. In ABI specifically, poor memory for one’s mood at the time of reporting it or the amount of effort required to regularly record mood may be too demanding.

Conversely, there was greater endorsement of activity scheduling by participants for enforcing a structure on one’s week, providing cognitive benefits such as enhanced memory, and helping sustain a good mood. Anticipatory benefits of enjoyable activities were noted to be mood enhancing, consistent with research demonstrating that individuals with ABI with depression have a reduced positive bias in prospective cognition [40]. Despite its benefits, some participants preferred spontaneously engaging in activities, and were hesitant about activity scheduling because of the difficulty of anticipating whether they would be in the “right mood” at the time. Possibly, spontaneously engaging in activities is protective – that is, the person may feel less emotionally invested in activities they have not spent effort in planning, potentially reducing the negative impact of failed activities. Whether encouraging spontaneity in BA increases activity level in ABI, relative to concretely scheduled activities, is yet to be determined. Too much spontaneity may be ineffective if the individual has a lack of support at home to help prevent spontaneous actions turning into impulsive behaviours, or if there are barriers to spontaneity in a positive way (e.g., if travel by car is required to complete the activity). No participants in this study lived alone, which may partially explain preference for spontaneity; likely any “excessive” spontaneity (i.e., risky decision-making or disinhibition) would be mitigated by a family member.

### BA techniques: Active Engagement, Alternative Solutions, and Self-Rewards

BA techniques to increase the likelihood of reinforcement included supporting active engagement, generating alternative solutions to planned activities, and self-rewards. Though alternative solutions could be perceived as too effortful, a noteworthy result was that participants made use of this technique if activities involved others. Possibly, this is representative of the Relation to Values theme, where participants felt that connecting with, or responsibility to, others was important even when effortful. Generating alternative solutions has been used in CBT in ABI as a means of improving flexible thinking [16] but for BA, which is predominantly behavioural, regular use of alternative solutions could make activities burdensome in general, and increase avoidance. This may be particularly relevant for ABI survivors with executive functioning difficulties. Additionally, mood may impact uptake of more complex BA techniques; in a systematic review, mood disorders and passive personality traits were related to poor uptake of goal setting in stroke [59].

Participants generally found learning how to self-initiate activities and reduce avoidance as beneficial. By contrast, there was less endorsement of the likely value of self-rewards. This may reflect beliefs about compassion toward oneself. Chywl and colleagues [60] report that those who viewed self-compassionate actions as irresponsible, indulgent, or leading to complacency were less likely to demonstrate care toward themselves in hypothetical emotionally challenging scenarios. Whether or not preference for self-rewards and its effect on reinforcement is determined by these beliefs is unclear. Additionally, the type of activity rewarded may be important – historically, self-rewards have been used for necessary activities that are not highly intrinsically reinforcing (e.g., conflict resolution [32]). Further, both severe depression and ABI may lead to reduced ability to experience pleasure [54], thus even if an individual is receptive toward using self-rewards, these may not be effective. Exploration on whether self-rewards improve behaviour change in ABI is warranted.

### Psychoeducational topics: Mindfulness and Social & Communication skills

In adults without ABI, extensions of BA with social and communication skills have been delivered successfully [32, 61]. Participants in our study felt that these skills could be particularly affected by ABI [62, 63] and that they are important facilitators to sustaining social activity engagement. This suggests that explicit components of an ABI BA programme devoted to developing social and communication skills could be useful.

Mindfulness, though perceived as useful, was viewed as difficult to achieve in practice. Mazzucchelli and colleagues [61] noted mixed feedback on the mindfulness component of their BA intervention, concluding that encouraging mindfulness toward activities that are already in one’s routine is preferable, rather than encouraging scheduling new reinforcing activities *and* learning to savour these new activities. More broadly, the concept of “staying in the moment” was thought by participants to enhance acceptance of post-ABI changes. Incorporating therapeutic options that facilitate acceptance such as Acceptance and Commitment Therapy into BA may be beneficial; a previous trial of BA in stroke noted that many struggled with accepting their post-injury life and were not ready to focus on increasing activity levels [27]. It is interesting to consider whether either training individuals to attend to the present moment during specific activities, or encouraging a more general mantra of staying “in the moment” would have differential effects in post-ABI adjustment.

### Psychoeducational topics: Routine vs Enjoyable Activities and Managing Uncertainty

Other potential additions to BA include encouraging a balance between non-reinforcing but necessary (“routine”) and potentially reinforcing (“enjoyable”) activities, and managing responses to uncertainty in planning. Some participants were not inactive – indeed, some felt overwhelmed by activities in their week. Like self-rewards, prioritising enjoyable activities may be considered indulgent – possibly even more so in those with depression [64]. Potentially, given the increase in prioritising and planning activities reported by participants, the limited cognitive capacity for activities is dedicated to those that “must” be completed. It is interesting to consider whether increasing pleasurable activities for individuals who view enjoyable activities as too indulgent would be beneficial, or if challenging such beliefs would be better suited to other therapeutic approaches.

When reducing avoidance in BA, individual responses to uncertainty may be relevant. Greater amounts of uncertainty are commonly reported post-stroke and often lead to significant distress [65, 66]. This risks establishing a cycle of worry and avoidance that participants both recognised and saw the merit of reducing. Though adaptations of BA for anxiety exist [67], the degree to which incorporating uncertainty management would increase activity levels is unknown. Graded exposure to activities with varying levels of perceived uncertainty may result in a client with ABI requiring additional reassurance, and in this instance alternative solutions and self-rewards for the activity may become useful.

Though all BA components were perceived positively by at least some participants, we cannot determine from our data whether it is the use of particular techniques, or a *general* reframing of attitudes toward activities, that most contributes to success in BA in ABI.

## Clinical implications

Given the variety of components that can be used in BA, tailoring the intervention to the client’s circumstances and preferences as a means of sustaining habitual activity engagement post-ABI may enhance outcomes [68]. It may be worth assessing whether more effortful techniques such as alternative solutions are necessary. For participants in early stages of ABI, or those with more severe cognitive and physical abilities, activities with fewer steps or shorter duration may be useful to prioritise. In longer courses of BA, using value-based approaches may be useful in early sessions to establish a routine, with later sessions introducing more complex topics such as social and communication skills. Some individuals may be averse to explicit consideration of how activities affect their mood, or may not have accepted changes as a result of their injury, in which case other therapeutic approaches may be preferred in combination with or instead of BA. Finally, whether acceptability or effectiveness of BA in ABI is enhanced by preferences expressed by participants, or varies with previous experiences of psychological therapy, should be investigated in future trials of BA in ABI.

## Limitations

As the strength of qualitative research is its focus on the individual perspective, it does not provide or prioritize estimates of how frequently a given view occurs. Because ABI is a highly heterogeneous condition, likely there are an array of needs and difficulties beyond those presented here that can also influence outcome from BA. Here, we instead have generated an initial framework that considers features that may be more likely to be acceptable for individuals with ABI undergoing BA. Participants were all White British and thus perceptions of BA components across different cultures may result in additional themes. Participants were from a clinical research panel, and all had sufficient communication skills to participate in an interview. Additionally, participants mainly had strokes or tumours, therefore interviews with other ABI aetiologies (e.g., traumatic brain injury, hypoxia) would be valuable. Finally, our sample had many participants with a postgraduate degree living in an affluent area in the UK, thus public health contributors to depression such as poverty may be relevant in other samples. Data from clinical settings and in a broader range of participants may produce additional themes.

## Conclusion

This study adds to the sparse literature relevant to delivering BA in ABI. Among the most relevant generated themes were the importance of considering the role of time post-ABI and how specific BA components align with this, readiness to engage in mood interventions, perceived effort required to implement various BA components, and the perceived value of planned activities.

## Supplementary Information

**Additional file 1.**

## Data Availability

The dataset supporting the conclusions of this article is available in the study-specific Open Science Framework repository: http://osf.io/btwg3 The corresponding author (TM) may be contacted with any data requests.

## Abbreviations

ABI: Acquired brain injury
TBI: Traumatic brain injury
CBT: Cognitive Behavioural Therapy
BA: Behavioural Activation
CCNRP: Cambridge Cognitive Neuroscience Research Panel
