# Supplementary material for: Developing behavioural activation for people with acquired brain injury: a qualitative interpretive description study of barriers and facilitators to activity engagement
Source: BMC Psychol. 2023 Jul 13;11:207. doi: 10.1186/s40359-023-01230-2 (PMC10339630; doi:10.1186/s40359-023-01230-2)
Supplement: Supplementary file 1 — Additional file 1. [file 40359_2023_1230_MOESM1_ESM.docx]

**Interview Script (Participant Version, Final Iteration)**

Italicised text indicates comments for the interviewer (AK) in facilitating discussion throughout the interview.

**Date:**

**Participant ID#:**

The purpose of this interview is to get a better idea of how people with a brain injury solve problems with day to day activities. I will be asking you questions about how you think, feel, and act during everyday activities. There are no right or wrong answers. I would like you to be as honest and open as you can in this interview, although you are always free to not answer questions if you do not want to.

There may be times when I ask you about things that you have already talked about in your earlier answers. So if we have already talked about a certain event that I am asking you questions about, we will just clarify your answer, and then move on.

Also, I will ask you throughout the interview if there is anything else you have to add to your answer. This is not because your answers are wrong, but because I want to make sure that I get all the important information from you.

Please let me know throughout the interview if it is not clear what I am asking.

As you know, the interview will take us about 1 hour. If you are feeling tired or would like to take a break at any time, please let me know. We will break for 5 minutes after 30 minutes into the interview.

Do you have any questions at this point?

Ok, let’s get started.

**Valued Activities**

First, can you tell me how, on a scale of 1 to 100, how important the below categories are to you? 100 would mean very important, and 1 would mean not at all important.

1. Work
2. Self-education/learning
3. Volunteering
4. Intimacy/relationships
5. Family
6. Friendship
7. Religion/Spirituality
8. Entertainment/Recreation
9. Health/Fitness

Is there anything else that I haven’t mentioned that you feel is really important to you?

**Topic 1: Overall Activity Engagement Experiences**

Okay great, now we are going to talk about overall activities. When I say this I mean day-to-day things such as chores, work, appointments, and the like.

First of all, I’d like you to list three things that you complete in a typical week and can generally do since your brain injury.

*(Prompt)*

This can be anything you like, such as doing the dishes or spending time with a family member.

Okay, great. Can you tell me what helps you get *motivated* on the activities you’ve mentioned?

*(If participant can’t think of any, give specific examples such as below)*

If you are going out of the house to get groceries, for example, one problem might be that the grocery store is far and you don’t think you have the time to get there if you walk. One way to help you get started is to think about other ways to get there, like by public transit. Does thinking about different ways to get there, like in this example, help you get started on what you need to do?

Is there anything else that helps you get motivated for your daily activities?

Great. Have you ever had any problems, related to your brain injury or not, that might stop you from doing what you like *(use specific examples that participants listed)*? What did you do, if anything, to get through these problems?

If these problems ever interrupted your activity all together, how did you feel when you realized you did not complete the activity?

*(Prompt)*

Do you think that *not* finishing this activity made you feel better/worse?

*(if this does not come up organically)*

Okay, great. Now, I would like you to tell me how you feel when you’ve completed an activity.

*(Prompts)*

Did finishing (participant’s specific example) make you feel happy, or good at what you are doing? Did it affect the rest of your day? Do you think that finishing this activity made you feel better/worse?

*(if this does not come up organically)*

Do you think that finishing this activity helped you in other or future activities?

**Topic 2: Engaging in Enjoyable Activities**

Now, we are going to talk about enjoyable activities specifically. I will ask a lot about “enjoyable activities” – when I say this, I mean anything that is pleasant or makes you feel good or happy. This can be something simple like calling your best friend or finishing a task at work, or big things like spending a day in London or travelling abroad.

Do you have any questions?

On average, how many enjoyable activities would you say you do in a week since your brain injury?

*(Prompt)*

Enjoyable activities can be something like reading a book, or watching your favourite show – it doesn’t need to be anything fancy, as long as you enjoy it.

Would you say being a part of pleasant or enjoyable activities are important for you? Why or why not?

Now, I’d like you to list three enjoyable activities that you would like to do in the next week.

Can you think of anything that might get in the way of starting these future activities *(input specific examples*), whether related to your brain injury or not?

*(For interviewer to note)*

Barriers:

Now that what do you think would help you get motivated for these activities (input specific examples)?

*(For interviewer to note)*

Strategies:

**Topic 3: Potential New Strategies**

Now, we are going to talk about different ways someone can get through problems that come up when trying to complete an activity. I will ask you for your thoughts about whether you think these will work for you specifically.

1. **Mood Monitoring**

Some people find that keeping track of how day-to-day activities make them feel helps them feel good about trying out new things. Specifically, people rate how happy they feel after completing an activity in order to get a better sense of what makes them feel good. Do you think keeping track of your mood in everyday activities will help you? Why or why not?

*(Example prompt, but ideally make use of specific examples given by participant)*

Say you just spent a day with your best friend. You make a note to be aware of how you feel after spending time with your friend, and notice that you are happy. Knowing that spending time with your best friends makes you feel happy, you try to meet with your friends more often. Do you think this would help motivate you to try out new and potentially enjoyable activities?

On a scale of 1 to 100, how useful do you think keeping track of your daily mood would be to you?

(*If strategy is already employed)*

On a scale of 1 to 100, how useful is keeping track of your daily mood to you?

1. **Activity Scheduling**

Some people like to keep a diary to keep track of what they have to do. Specifically, sometimes someone will plan in pleasant activities to make sure they have activities to look forward to. Do you think keeping an activity diary will help you? Why or why not?

*(Example prompt, but ideally make use of specific examples given by participant)*

Let’s say you make sure to plan in a movie night at home every once in a while no matter what. Do you think this would be helpful for you?

On a scale of 1 to 100, how useful do you think keeping an activity diary would be to you?

(*If strategy is already employed)*

On a scale of 1 to 100, how useful is keeping an activity schedule is to you?

1. **Brainstorming Alternativee Solutions**

Sometimes it’s helpful to think ahead of time of different ways to tackle problems when trying out new or different activities. Specifically, it helps some people to think of what they usually do when faced with a problem, and then list other ways to solve it. Do you think this would help you solve any problems you might have starting an activity? Why or why not?

*(Example prompt, but ideally make use of specific examples given by participant)*

Let’s say that every time you want to go shopping, you are worried that your family member will be too busy to drive you, so you decide not to go shopping. The current way that this problem is “solved” is by not going shopping, even though it is something you want to do. Beforehand, you might think of different ways to get you to the store such as calling a taxi, taking public transportation, calling a friend, or even online shopping. Do you think this would be helpful?

One a scale of 1 to 100, how useful do you think listing different solutions to problems would be to you?

(*If strategy is already employed)*

On a scale of 1 to 100, how useful is thinking about back up plans ahead of time to you?

1. **Balancing Types of Activities**

Sometimes, people feel that they have too many stressful or important activities on the go, such as various appointments or job requirements. Some people find it helpful to plan pleasant activities to balance out the stressful activities. Do you think this will be helpful for you? Why or why not?

*(Example prompt, but ideally make use of specific examples given by participant)*

Let’s say you have a very busy week and it leaves you feeling frustrated. You decide Tuesdays will be your “relaxation days” where you spend your evening with your family to help you feel better. Do you think this would be helpful?

On a scale of 1 to 100, how useful do you think balancing mandatory and pleasant activities would be to you?

(*If strategy is already employed)*

On a scale of 1 to 100, how useful is balancing enjoyable and mandatory activities to you?

1. **Active Engagement**

Sometimes, it is easier to let things happen to you rather than make things happen. Some people feel that that playing an active role in their life helps build their confidence and control their lives. Do you think this would be helpful for you? Why or why not?

*(Example prompt, but ideally make use of specific examples given by participant)*

Let’s say that you want to talk to your cousin who lives across the country, but you don’t want to bother them so you wait for them to call. An active way of dealing with this situation would be to call them anyway to start the conversation. Do you think learning to do this would be helpful for you?

On a scale of 1 to 100, how useful do you think dealing with situations actively would be for you?

(*If strategy is already employed)*

On a scale of 1 to 100, how useful is taking initiative to plan activities to you?

1. **Mindfulness**

Some people like to remind themselves to stay “in the moment” – that is, being aware of what is happening right now rather than looking back to the past or thinking about what will happen in the future. This helps some people get the most out of what they are doing and enjoy each day more. Do you think this would be helpful for you? Why or why not?

*(Example prompt, but ideally make use of specific examples given by participant)*

Let’s say you are having tea with your friends. You keep on worrying about tomorrow, when you have to visit the doctor’s, call the hydro company, and get some food for a family dinner. This distracts you from the present moment, which is tea with your friends. In mindfulness, you would learn to keep yourself focused on tea with friends rather than tomorrow’s worries. Do you think this would help you?

On a scale of 1 to 100, how useful do you think mindfulness would be for you?

(*If strategy is already employed)*

On a scale of 1 to 100, how useful is mindfulness to you?

1. **Communication and Social Skills**

Sometimes, people have a hard time telling others what they want to do, or don’t know how to start a conversation about a difficult subject. Other times, we can feel uncertain about speaking with new people. It is helpful for some people to learn how to better communicate with others so they can say what’s on their mind or meet new people. Do you think this would be helpful? Why or why not?

*(Example prompt, but ideally make use of specific examples given by participant)*

Let’s say you are trying to tell your partner that you’d like to spend a day at home solving puzzles rather than go to the park, but your partner insists that the park is a good idea. Rather than tell them what you want to do, you go along with it even though you don’t want to. Learning how to bring up this information in a respectful way can help avoid hurting any feelings. Do you think this would be helpful?

On a scale of 1 to 100, how useful do you think working on your communication skills would be for you?

(*If strategy is already employed)*

On a scale of 1 to 100, how useful are communicating to others about difficult topics to you?

1. **Managing Uncertainty**

With any health condition or disability, there can be a lot of uncertainty about how you will handle a new situation. For some people, the uncertainty is too worrisome and makes them feel anxious. It can be helpful to learn how to cope with uncertain situations in order to feel more comfortable when starting a new activity. Do you think this will be helpful for you?

*(Example prompt, but ideally make use of specific examples given by participant)*

Let’s say your family wants to take you to shopping in Norwich. You have not yet been to Norwich, and you currently use a walker to help you get around. You are unsure whether the pavement will be uneven, whether all of the shops will have appropriate disabled access, and if you can manage a day in Norwich. These worries make you not want to go to Norwich. Some people find it helpful to manage uncertain situations by taking part in similar uncertain experiences in order to prepare themselves, like going to see a film without knowing anything about it. Do you think this would be useful for you?

On a scale of 1 to 100, how useful do you think working on managing uncertainty would be for you?

(*If strategy is already employed)*

On a scale of 1 to 100, how useful is managing uncertainty to you?

1. **Social Skills (no longer used after October 2018 version of interview)**

Every once in while, people can feel uncomfortable or awkward speaking with friends or family, and lose confidence in their social skills. This can either be something people feel generally uncomfortable with, or it could be something that people suddenly are unsure about after their injury. This can lead them to keep to themselves and avoid new activities that involves other people. Some people find it useful to practice their social skills with other people in order to become more confident. Do you think this would be useful for you?

*(Example prompt, but ideally make use of specific examples given by participant)*

Let’s say you are going to visit one of your friends. Your friend is bringing some people that she knows from her neighbourhood. You have not met them yet, and you are unsure if they know that you have had a brain injury. You suddenly feel uncomfortable about meeting new people because you don’t know how they will react to you, and you then decide not to go to your friend’s home. When learning how to improve social skills, you would practice interacting with new people on a regular basis in order to feel more comfortable. Do you think this would be useful for you?

On a scale of 1 to 100, how useful do you think working on your social skills would be for you?

(*If strategy is already employed)*

On a scale of 1 to 100, how useful are your social skills to you?

Is there anything else that you think would be useful in helping you engage in enjoyable activities?
